# Co-treatment of gonadotropin and letrozole in infertile women with endometriosis: A double-blind randomized clinical trial

**DOI:** 10.18502/ijrm.v20i6.11444

**Published:** 2022-07-06

**Authors:** Mahbod Ebrahimi, Firoozeh Akbari Asbagh, Fatemeh Davari Tanha, Hamideh Pakniat, Elham Feizabad, Yasin Rasouli

**Affiliations:** ^1^Department of Obstetrics and Gynecology, Yas Hospital, Tehran University of Medical Sciences, Tehran, Iran.; ^2^Department of Obstetrics and Gynecology, Faculty of Medicine, Qazvin University of Medical Sciences, Qazvin, Iran.; ^3^Faculty of Pharmacy, University of Szeged, Hungary.

**Keywords:** Gonadotropin-releasing hormone, Fertilization in vitro, Letrozole, Endometriosis.

## Abstract

**Background:**

The common causes of infertility in women with endometriosis are folliculogenesis alternation, steroidogenesis and fertilization impairment, oocyte and embryo quality reduction, and implantation defect.

**Objective:**

To compare in vitro fertilization (IVF) cycle success rates of women with endometriosis who were treated with letrozole + gonadotropin (LA) vs. placebo + gonadotropin (PA).

**Materials and Methods:**

This double-blind, randomized clinical trial study was conducted with 94 infertile women with endometriosis (47 in the LA group and 47 in the PA group) who were candidates for IVF, from April-June 2021. For all participants, the long agonist protocol was applied. In both groups, gonadotropin-releasing hormone agonist was prescribed in the mid-luteal stage and from the third day of the cycle, and gonadotropin was started and its doses were regulated based on the patient's age, serum anti-Mullerian hormone and follicle-stimulating hormone. From the third day of the menstrual cycle, 5 mg of letrozole daily for 5 days was prescribed for the LA group, while the placebo was prescribed for the PA group on the identical days and duration. After embryo transfer, biochemical and clinical pregnancy were measured in the 2 groups.

**Results:**

The gonadotropin dosage (p < 0.01) and estradiol level (p = 0.02) on the human chorionic gonadotropin administration day were significantly lower in the LA group compared with in the PA group. Fetus transfer was done for 32 women. No significant differences were detected between the study groups regarding biochemical or clinical pregnancy (p = 0.72 for both).

**Conclusion:**

Letrozole as a co-treatment drug in the IVF cycle of women with endometriosis can significantly reduce the gonadotropin dosage and estradiol level with the same pregnancy rates.

## 1. Introduction

The prevalence of endometriosis, tissue and glands of endometrium placed outside the uterus, in infertile women is 25-50%, and it can cause the failure of in vitro fertilization (IVF) treatment (1, 2). The usual causes of infertility in women with endometriosis are folliculogenesis alternation, steroidogenesis and fertilization impairment, oocyte and embryo quality reduction, implantation defect, and pelvic adhesions (3). Endometriosis is an estrogen-dependent disease associated with increased aromatase enzyme expression and concentration and some pathologic mediator secretion, such as of estradiol and prostaglandin E ${}_{2}$ (1). These pathologic mediators have an important role in promoting the growth and invasion of endometriotic tissue, pain, inflammation, and infertility (4).

Nowadays, assisted-reproductive technology can have a considerable role in resolving infertility problems in most couples. However, previous studies have shown significantly lower successful fertilization rates in endometriosis rather than in other causes of infertility in IVF cycles (5-7). Letrozole is a selective aromatase inhibitor, that causes a decrease in the estrogen concentration. The result of a decline in estrogen level is an increase in follicle-stimulating hormone secretion, ovarian follicle-stimulating hormone receptor affinity, antral follicle growth, follicle phase enhancement, and follicle development. The other effect of letrozole is reducing estradiol and prostaglandin E ${}_{2}$ production, which affects oocyte quality (8, 9). Letrozole has been recommended in some research for improving fertility results in poor responder women, treatment of endometriosis-related pelvic pain, treatment of hormone receptor-positive breast cancer, and fertility preservation in women with breast cancer (10-12).

However, studies about the application of letrozole in the IVF cycles of women with endometriosis are rare and more studies are needed on this topic. This study was designed to compare IVF cycle success rates of women with endometriosis treated with letrozole + gonadotropin (LA) vs. placebo + gonadotropin (PA).

## 2. Materials and Methods 

This double-blind, randomized clinical trial study with a parallel design was done with 94 infertile women with endometriosis referred to our IVF Unit at Yas hospital, Tehran, Iran from April-June 2021. The inclusion criteria included women with pelvic endometriosis and primary infertility, in their first IVF cycle, 18-35 yr old, body mass index < 30 kg/m^2^, serum anti-Mullerian hormone (AMH) > 1 ng/ml, and partner sperm motility of at least 20%.

Women who had undergone letrozole or clomiphene therapy with the aim of inducing ovulation, or who had deeply infiltrating endometriosis, or submucosal or intramural myoma, detected in transvaginal ultrasound (TVS), or with uterine diseases were excluded.

This study was conducted double-blind. The participants, because of placebo usage, did not know the type of their treatment. Also, the analyzer did not know about the treatment group codes in the analysis data sheet.

Using random allocation, participants were divided by the corresponding author into the 2 groups of LA and PA (n = 47/each). First, 47 letter As and 47 letter Bs were written on papers without other markings. All of the papers were placed in a bag, and for each woman, a paper was taken randomly and without replacement. In addition, interventions A and B were randomly assigned to the LA group and PA group, respectively.

For all participants, the long agonist protocol was applied. 300 mcg of gonadotropin-releasing hormone (GnRH) agonist (CinnaFact, CinnaGen Company, Iran) was prescribed in the mid-luteal stage (7 days before the anticipated menstruation). Then, on the third day of menstruation, the women were evaluated with TVS (4.5-7 MHz vaginal probe, Sono line G-40, Siemens, Germany) for endometrial thickness (ET) and antral follicle count assessment in both ovaries.

In both groups, from the third day of the cycle, gonadotropin (CinnalF, CinnaGen Company, Iran) was started and its doses were regulated based on the patient's age, serum AMH and follicle-stimulating hormone. From the third day of the menstrual cycle, 5 mg of letrozole daily for 5 days was prescribed for the LA group, while the placebo was prescribed for the PA group on the identical days and duration.

Repeated TVS examinations were done with the aim of follicular maturation assessment. Human menopausal gonadotropin (Pooyesh Daru, Iran) was added whenever follicle(s) sizes were ≥ 10-12 mm. furthermore, CinnalF continued until the triggering day of ovulation.

Then, 250 µg of choriogonadotropin alfa (Ovitrelle, Merck Serono, Italy) was administrated subcutaneously if at least 2 follicles with ≥ 18 mm in diameter were reported and serum estradiol concentration (on trigger day) was ≥ 500 pg/mL. The cycle was cancelled when the above criteria were not detected after 10-12 days following stimulation.

After 34-36 hr following choriogonadotropin alfa initiation, oocyte retrieval with aid of TVS (Honda Company, Japan) was conducted under spinal anesthesia. Then, for all the cycles, intracytoplasmic sperm injection was carried out.

Fresh embryo transfer was done for all of the participants unless there were contraindications such as ovarian hyperstimulation syndrome, pelvic and abdominal pain due to endometriosis, endometrioma necessitating surgery, or at the woman's request. In these mentioned conditions, frozen embryo transfer was done.

In fresh embryo transfer, 100 mg of progesterone (Iran Hormone Company, Iran) was injected daily immediately after oocyte retrieval and for 2 wk. 3 days after puncture day, embryos (in cleavage form) were transferred. Serum β-human chorionic gonadotropin (β-hCG) was checked on the 14 ${}^{\text{th}}$ day of embryo transfer, and if pregnancy was confirmed, 400 mg of suppository tablets of progesterone (Cyclogest, Actavis, Barnstaple, UK) was initiated and continued daily to the end of pregnancy.

In frozen embryo transfer, from the third day of the menstrual cycle, 6 mg of estradiol (Abu Reihan Pharmaceutical Company, Iran) was given daily as an oral pill. ET was assessed serially each 3-4 days, and when ET was > 8 mm, 100 mg of progesterone (Iran Hormone Company, Iran) was injected, and continued daily. After 4 days, progesterone-initiated embryos (in cleavage form) were transferred. Serum β-hCG was checked on the 14 ${}^{\text{th}}$ day after embryo transfer, and if pregnancy occurred, 400 mg of suppository tablets of progesterone (Cyclogest, Actavis, Barnstaple, UK) were given daily with estradiol until the end of pregnancy.

The following data were recorded for both groups: age, marriage and infertility duration, body mass index, thyroid-stimulating hormone, prolactin, AMH, and follicle-stimulating hormone.

The total prescribed dosage of gonadotropin (calculated on the trigger day), the serum estradiol level (measured on the trigger day), the oocyte number and quality (determined on the oocyte retrieval day according to the oocyte maturity grading), and the embryo quality (categorized based on the Gardner morphological assessment system, which grades expansion status, inner cell mass from A-C, trophectoderm from A-C, and blastocyst growth stage from 3-6) (13), were analyzed for all patients.

Serum β-hCG was measured on the 14 ${}^{\text{th}}$ day after embryo transfer (to determine biochemical pregnancy), and pregnancy sac observation through TVS was assessed 6 wk after embryo transfer (to determine clinical pregnancy).

### Ethical considerations

This study was approved by the Ethics Committee of Sina hospital, affiliated to Tehran University of Medical Sciences, Tehran, Iran (Code: IR.TUMS.SINAHOSPITAL.REC.1399.100). The study was registered in the Iranian randomized clinical trial registry and was done in compliance with the Declaration of Helsinki and all the participants signed an informed consent form.

### Statistical analysis

All of the statistical analyses were done using the Statistical Package for the Social Sciences (SPSS) version 24.0. P-values < 0.05 were considered statistically significant. The independent *t* test and non-parametric Mann-Whitney U test were used to evaluate the differences in means. A Chi-square test and Fisher's exact test were applied to assess the differences in proportions.

## 3. Results

**Figure 1:**
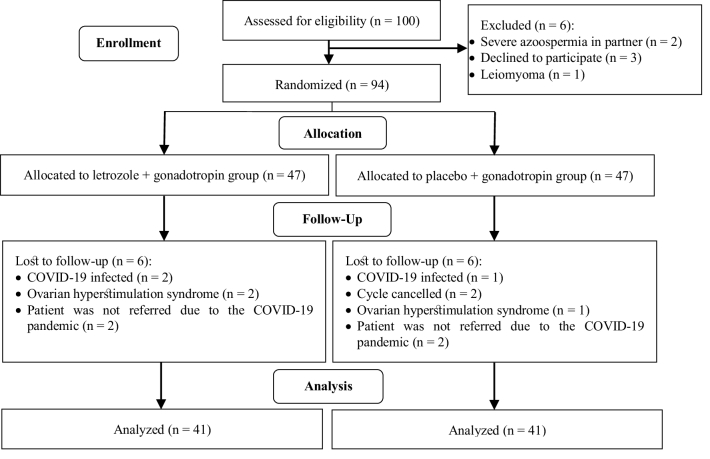
Consort flow diagram of women with endometriosis who underwent letrozole and gonadotropin treatment.

100 infertile women were assessed for eligibility; of them, 6 women were excluded due to: severe azoospermia in their partners (n = 2), leiomyoma (n = 1), and declined to participate (n = 3). In total, 94 women were randomized to the LA and PA groups. During the study, 12 participants (6 from each group) did not refer for follow-up and were considered lost to follow-up. Finally, data from 82 women with endometriosis were analyzed (Figure 1).

The mean age and infertility duration were 31.49 ± 4.64 yr and 5.01 ± 3.37 yr, respectively. The basic characteristics of participants did not differ significantly between the 2 study groups (Table I).

Gonadotropin dosage (p < 0.01) and estradiol level (p = 0.02) on the hCG administration day were significantly lower in the LA group in comparison with the PA group. The other cycle characteristics did not differ significantly between the 2 study groups (Table II).

Embryo transfer was done for 32 women. Positive ß-hCG (indicating biochemical pregnancy) was observed in 17 women (53.1%), and clinical pregnancy in 13 (40.6%) women. No significant differences were detected between the study groups regarding biochemical or clinical pregnancy (p = 0.72 for both). The frequency of pregnancies according to embryo transfer type are summarized in table III.

**Table 1:** The basic characteristics of participants (n = 41/each)


**Variables**	**PA group**	**LA group **	**P-value**
**Age (yr)**	31.31 ± 4.45	31.66 ± 4.87	0.71*
**Marriage duration (yr)**	7.08 ± 4.17	6.75 ± 4.25	0.70*
**Infertility duration (yr)**	5.13 ± 3.21	4.88 ± 3.55	0.73*
**Body mass index (kg/m^2^)**	24.44 ± 2.69	25.1 ± 2.63	0.24*
**TSH (mIU/L)**	2.59 ± 0.98	2.30 ± 0.85	0.14**
**PRL (ng/mL)**	18.00 ± 4.96	18.26 ± 6.91	0.83**
**FSH (mIU/mL)**	5.38 ± 2.30	6.19 ± 2.47	0.11*
**AMH (ng/ml)**	2.90 ± 0.30	3.30 ± 0.24	0.31*
**Baseline ET (mm)**	3.23 ± 0.33	3.37 ± 0.51	0.14*
**Baseline AFC (n)**	5.97 ± 1.27	6.08 ± 1.94	0.74*
Data presented as Mean ± standard deviation. *Mann-Whitney U test, **Independent *t* test. PA: Placebo + gonadotropin group, LA: Letrozole + gonadotropin group, TSH: Thyroid-stimulating hormone, PRL: Prolactin, FSH: Follicle-stimulating hormone, AMH: Anti-Mullerian hormone, ET: Endometrial thickness, AFC: Antral follicle counts			

**Table 2:** Comparison of cycle characteristics in the 2 study groups (n = 41/each)


**Variables**		**PA group**	**LA group**	**P-value**
ET (mm)*		7.51 ± 1.05	7.80 ± 1.14	0.20 ${}^{†}$
AFC (n)*		8.88 ± 3.05	9.28 ± 3.50	0.56 ${}^{†}$
Gonadotropin (dose)*		3228.88 ± 944.49	2537.22 ± 952.72	0.00 ${}^{†}$
Gonadotropin (day of prescription)*		10.82 ± 2.02	10.15 ± 1.39	0.07 ${}^{†}$
HMG (day of prescription)*		5.17 ± 2.00	5.13 ± 1.39	0.90 ${}^{†}$
Estradiol (day of hCG)*		2604.51 ± 1823.36	1837.62 ± 1181.69	0.02 ${}^{†}$
Oocyte number*		9.20 ± 3.17	10.40 ± 5.50	0.20 ${}^{†}$
**Oocyte quality**				
	Germinal vesicle**	34 (75.6)	34 (75.6)	
	Metaphase I**	10 (22.2)	6 (17.8)	
	Metaphase II**	1 (2.2)	5 (6.7)	0.16 ${}^{††}$
**Embryo quality**				
	A**	33 (73.3)	33 (73.3)		
	B**	11 (24.4)	11 (24.4)	
	C**	1 (2.2)	1 (2.2)	0.10 ${}^{†††}$
*Data presented as Mean ± standard deviation. **Data presented as n (%). Data were compared by ${}^{†}$ Mann-Whitney U test, ${}^{††}$ Fisher's exact test, ${}^{†††}$ Chi-square test. PA: Placebo + gonadotropin group, LA: Letrozole + gonadotropin group, ET: Endometrial thickness, AFC: Antral follicle count, HMG: Human menopausal gonadotropin, hCG: Human chorionic gonadotropin				

**Table 3:** The frequency of pregnancies according to embryo transfer type


**Variables **	**Fresh embryo transfer**		**Frozen embryo transfer**		
**Group name**	**PA (n = 8)**	**LA (n = 3)**	**P-value**	**PA (n = 8)**	**LA (n = 13)**	**P-value**
**Biochemical pregnancy**	4 (50.0)	2 (66.6)		1.00	5 (62.5)		6 (46.1)	0.66
**Clinical pregnancy**	2 (25.0)	2 (66.6)	0.49	5 (62.5)	4 (30.7)	0.20
Data presented as n (%). Fisher's exact test was used. PA: Placebo + gonadotropin group, LA: Letrozole + gonadotropin group						

## 4. Discussion

Our study showed that gonadotropin dosage was significantly lower in the LA group compared to the PA group. Furthermore, the estradiol level on the hCG administration day was significantly lower in the LA group in comparison with the PA group. However, no significant differences were detected between the study groups regarding biochemical or clinical pregnancy.

Infertile women with endometriosis have a poorer response to infertility treatment than women with other causes of infertility; the main reasons may be a reduction in embryo quality, endometrial reception and implantation rates, and enhancement in inflammation and aromatase synthesis (14-16).

Previous research has attempted to find solutions to counter the adverse impact of infertility in women with endometriosis, such as by evaluating the effect of a post-operative systematic GnRH agonist prescription (17, 18) and pre-treatment with GnRH agonist (19); however, this problem remains unresolved, and further studies are needed.

This study assessed the effect of a combination of letrozole and gonadotropin compared with gonadotropin alone on the outcomes of IVF treatment in infertile women with endometriosis. The results showed that this combination was associated with significantly lower estradiol levels and dose of Cinnal-f. However, it had no significant influence on oocyte number or quality, embryo quality, biochemical pregnancy, or clinical pregnancy rates.

Our results showing that letrozole application was associated with a significantly lower dose of gonadotropin and estradiol level are in line with studies conducted on normal responders (20), women with breast cancer (21), and poor responders (22, 23). However, other studies found that the gonadotropin dose was similar in the letrozole vs. control groups that they examined (12, 24); this could be due to the lack of randomization in these studies and because they enrolled women with laparoscopic-approved endometriosis.

Some studies (2, 12, 20-22), similarly to this study, indicated that adding letrozole to the IVF regimen had no significant effect on stimulation duration, oocyte number, or embryo number or quality. In contrast, in other studies, letrozole was able to significantly affect the number and quality of oocytes (23, 24) and the length of stimulation (23, 25).

Although some previous studies (2, 20-24), in line with our study, indicated no significant variation in pregnancy proportions with letrozole application, Piedimonte et al. (25) indicated that pregnancy and live-birth rates increased significantly following administration of a letrozole and GnRH agonist combination. Given the varied findings of previous studies, future studies seem needed to assess the effect of varying doses of letrozole in infertile women with endometriosis, as well as to compare the effect of a combination of letrozole with agonist vs. antagonist IVF protocol.

### Limitation

The small sample size was a limitation of our study.

## 5. Conclusion

According to the study findings, using letrozole as a co-treatment drug in the IVF cycle of women with endometriosis can significantly reduce gonadotropin dosage and estradiol level with the same pregnancy rates.

##  Conflict of Interest

The authors declare that there is no conflict of interest.
